# Are we there yet? CAR‐T therapy in multiple myeloma

**DOI:** 10.1111/bjh.19896

**Published:** 2024-11-19

**Authors:** Eitan Mirvis, Reuben Benjamin

**Affiliations:** ^1^ School of Cancer & Pharmaceutical Sciences, King's College London London UK; ^2^ Department of Haematology King's College Hospital NHS Foundation Trust London UK

**Keywords:** cellular therapies, multiple myeloma, myeloma therapy

## Abstract

The last few years have seen a revolution in cellular immunotherapies for multiple myeloma (MM) with novel antigen targets. The principle new target is B‐cell maturation antigen (BCMA). Autologous chimeric antigen receptor T‐cell (CAR‐T) therapy directed against BCMA was first approved by the US Food and Drug Administration (FDA) and European Medicines Agency (EMA) in 2021, although approval by the National Institute for Health and Care Excellent (NICE) is awaited. Initial response rates in patients with heavily pretreated MM have been impressive, but patients are still relapsing. Furthermore, CAR‐T manufacturing is expensive and time‐consuming, and T‐cell fitness is impaired by prior MM treatment. Numerous strategies to improve outcomes and delivery of cellular immunotherapy are under investigation, including next‐generation CARs, allogeneic ‘off‐the‐shelf’ CARs and targeting of other MM antigens including G protein‐coupled receptor, class C, group 5, member D (GPRC5D), Fc receptor homologue 5 (FcRH5), cluster of differentiation (CD)19, signalling lymphocyte activation molecule family member 7 (SLAMF7) and several others. In this exciting and rapidly evolving treatment landscape, this review evaluates the most recent clinical and preclinical data pertaining to these new cellular immunotherapies and explores strategies to overcome resistance pathways. On the protracted journey to a long‐term cure, we outline the challenges that lie ahead and ask, ‘Are we there yet?’

## Graphical abstract

Chimeric antigen receptor T‐cell (CAR‐T) therapy has achieved hitherto unseen responses in heavily pretreated relapsed/refractory multiple myeloma. Nevertheless, patients still relapse. Mechanisms of resistance to CAR‐T include tumour‐intrinsic factors, the immunosuppressive tumour microenvironment, impaired baseline T‐cell fitness and CAR‐T factors. To improve outcomes, multifarious strategies to overcome CAR‐T resistance are in development. Created in BioRender. Mirvis, E. (2024) https://BioRender.com/h19e684.

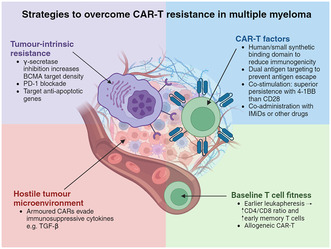

## INTRODUCTION

Multiple myeloma (MM) is a clonal plasma cell (PC) neoplasm characterised by features of end‐organ damage including hypercalcaemia, renal insufficiency, anaemia and lytic bone lesions.^1^ Median overall survival (OS) ranges from 7 to 10 years in standard‐risk patients to 3 years in patients with high‐risk cytogenetic abnormalities such as t(4;14), t(14;16), t(14;20) and del(17/17p).^2^ In the MAMMOTH study, median OS was only 9.2 months in patients triple refractory to a proteasome inhibitor (PI), immunomodulatory drug (IMiD) and anti‐cluster of differentiation (CD)38 monoclonal antibody (CD38 mAb).^3^ Second autologous stem cell transplant (ASCT) has limited efficacy; 975 patients salvaged with second ASCT had cumulative incidence of relapse/progression of 49% at 1 year and 84% at 3 years.^4^ Outcomes of allogeneic SCT in MM are generally poor, with 1‐year transplant‐related mortality at 23.5% and 5‐year OS at 22.2%.^5^ There is, therefore, an urgent need for treatment with long‐term curative potential for relapsed/refractory MM (RRMM).

Novel therapeutic strategies include bispecific T‐cell engagers (BiTEs) and chimeric antigen receptor T‐cell (CAR‐T) therapy. BiTEs consist of two antigen recognition domains connected via a linker. They create an indirect immunological synapse between MM cells and T‐cells, resulting in T‐cell activation and MM cell death.^6^ BiTEs targeting B‐cell maturation antigen (BCMA), G protein‐coupled receptor, class C, group 5, member D (GPRC5D) and Fc receptor homologue 5 (FcRH5) have been developed and are reviewed elsewhere.^7^, ^8^, ^9^

CAR‐T therapy involves reprogramming T cells with a CAR construct targeting a tumour‐associated antigen. Antigen binding triggers CAR‐T activation, proliferation and cytotoxic effector functions. First approved in 2017, CD19 CAR‐T now plays a transformative role in relapsed/refractory B‐cell acute lymphoblastic leukaemia, large B‐cell lymphomas and mantle cell lymphoma.^10^ More recently, CARs have been manufactured against MM tumour antigens, including BCMA, GPRC5D and several others.

In addition to an intracellular CD3ζ signal transduction domain, second‐generation CARs are equipped with a costimulatory domain, for example, CD28 or 4‐1BB, which improves proliferation, cytokine secretion, resistance to apoptosis and persistence.^11^ All currently licensed CARs are second generation. Third‐generation CARs have two costimulatory domains, usually CD28 combined with 4‐1BB. Fourth‐/next‐generation CARs have further modifications, including armoured CAR‐T cells, which overpower the inhibitory tumour microenvironment (TME), and T cells redirected for universal cytokine‐mediated killing (TRUCKs), which express cytokines to promote CAR‐T function and survival.^12^

CAR‐T manufacture begins with leukapheresis; T cells are subsequently activated and transduced with a viral vector encoding the CAR, resulting in CAR expression on the T‐cell surface. Following CAR‐T expansion, the product is concentrated and cryopreserved.^13^ Prior to CAR‐T infusion, the recipient requires lymphodepletion, usually with fludarabine and cyclophosphamide.^14^

The most common CAR‐T toxicity is cytokine release syndrome (CRS), a state of immune over‐activation associated with CAR‐T expansion. Grade 1 CRS involves fever; however, symptoms can rapidly progress to multiorgan failure. In addition to supportive care, severe CRS is treated with tocilizumab (anti‐IL‐6 mAb) and if refractory, corticosteroids. Immune effector cell‐associated neurotoxicity syndrome (ICANS) can range from mild confusion to seizures to fatal cerebral oedema. The mechanism is poorly understood.^15^ Other common toxicities include cytopaenias and infection.

This review will discuss the novel cellular immunotherapy targets being explored in RRMM, with an emphasis on strategies to overcome resistance and improve depth and duration of response.

## NOVEL IMMUNOTHERAPY TARGETS

### BCMA

**FIGURE 1:**
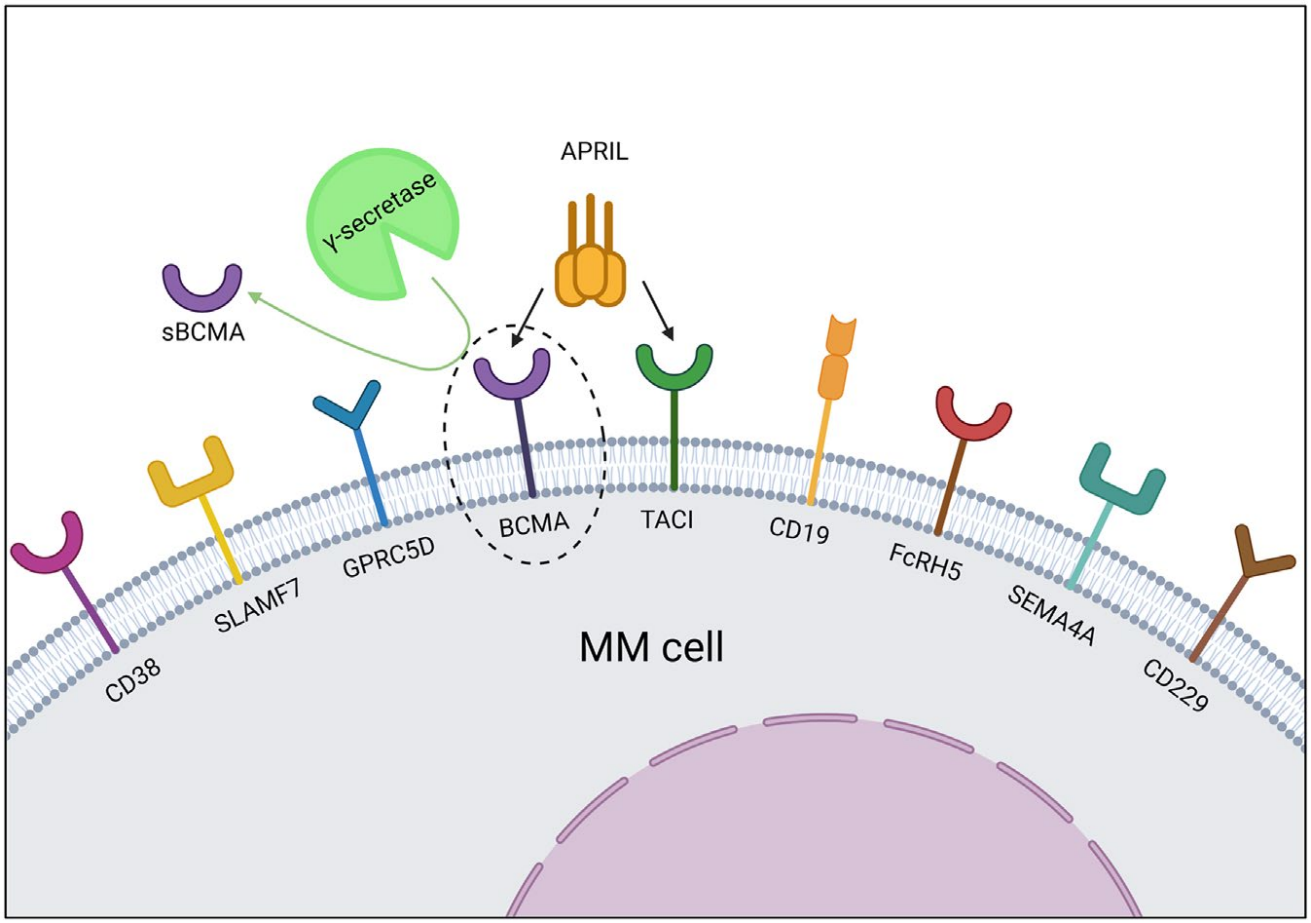
Novel cellular immunotherapy targets in multiple myeloma (MM). The γ‐secretase complex cleaves B‐cell maturation antigen (BCMA) from the MM cell surface, releasing soluble BCMA (sBCMA) into the bloodstream. APRIL is one of the two natural ligands for BCMA and TACI. Created in BioRender. Mirvis, E. (2024) https://BioRender.com/r05s346.

BCMA (CD269) is a pro‐survival non‐tyrosine kinase receptor surface glycoprotein and a member of the tumour necrosis factor receptor (TNFR) superfamily (Figure 1). Expression is restricted to late memory B cells and PCs and is highest in MM cells. BCMA transmits downstream signals which are important for PC survival.^16^

BCMA CAR‐T has shown impressive results in heavily pretreated MM. A meta‐analysis of 21 trials including total of 761 RRMM patients showed overall response rate (ORR) of 87%, complete response rate (CRR) of 44%, median progression‐free surviv (PFS) of 8.77 months and median OS of 18.87 months.^17^ Idecabtagene vicleucel (Ide‐cel) contains a murine anti‐BCMA single‐chain variable fragment (scFv) with 4‐1BB as the costimulatory domain delivered via a lentiviral vector. In the phase II KarMMa study, 128 patients who progressed after median of six lines of treatment (LOT) attained ORR of 73% and median PFS of 8.8 months (Table 1).^19^ There was correlation between depth and duration of response, with duration of 4.5, 10.4 and 19 months for patients attaining partial response (PR), very good PR (VGPR) and CR/stringent CR (sCR) respectively. In phase III KarMMa‐3, which enrolled patients treated with two to four prior LOT, Ide‐cel achieved ORR of 71% compared with 42% in the standard chemotherapy treatment arm; median PFS was 13.3 months versus 4.4 months. Grades 3–4 CRS occurred in 5% and grades 3–4 ICANS in 3% (Table 2).^21^ A retrospective study of real‐world data included 196 patients, 75% of whom would have been ineligible for KarMMa due to comorbidities. ORR was 84%, median PFS 8.5 months and median OS 12.5 months.^47^ The largest real‐world study of Ide‐cel outcomes includes 603 patients with median of seven prior LOT. ORR was 71%, again comparable with KarMMa and KarMMa‐3. With median follow‐up of 6.6 months, estimated 6‐month PFS was 62% and OS of 82%.^48^ Multivariate analysis has identified prior BMCA‐directed therapy, high‐risk cytogenetics and elevated baseline ferritin level as potential predictors of early progression/refractory disease following Ide‐cel.^49^ KarMMa‐2 (NCT03601078) is investigating Ide‐cel in clinical high‐risk MM patients at an earlier stage of the disease. Interim results demonstrate ORR of 83.8% and median PFS of 11.4 months in patients who progressed within 18 months of initial ASCT.^20^

**TABLE 1:** CAR clinical trials.

Reference	Target	Agent, source	Auto/allo	Phase	Prior LOT (median)	Enrolment (*n*)	ORR (%)	Median PFS (months)	Median OS (months)	CRS grade ≥3 (%)	ICANS grade ≥3 (%)
NCT02658929 Raje et al^18^	BCMA	Ide‐cel, murine	Auto	I	3–14 (7)	33	85	11.8	NR	6	3
NCT03361748 KarMMa^19^	BCMA	Ide‐cel, murine	Auto	II	3–16 (6)	140	73	8.8	NR	5	3
NCT03601078 KarMMa‐2 Cohort 2A^20^	BCMA	Ide‐cel, murine	Auto	II	1 ± bridging	39	83.8	11.4	NR	2.7	0
NCT03651128 KarMMa‐3^21^	BCMA	Ide‐cel, murine	Auto	III	2–4 (3)	386	71 *vs* 42	13.3 *vs* 4.4	NR	5	3
NCT03548207 CARTITUDE‐1^22^	BCMA	Cilta‐cel, camelid	Auto	Ib/II	≥3 (6)	113	97	34.9	NR	4	9
NCT04133636 CARTITUDE‐2 Cohort B^23^	BCMA	Cilta‐cel, camelid	Auto	II	1	18	88.9	NR	NR	5.6	0
NCT04181827 CARTITUDE‐4^24^	BCMA	Cila‐cel, camelid	Auto	III	1–3 (2)	419	84.6 *vs* 67.3	NR *vs* 11.8	NR	1.1	0
NCT05243212 Magen et al^25^	BCMA	CAR‐BCMA, murine	Auto	Ib/II	3–5 (4)	32	59	NR	NR	3	0
Mikkilenini et al^26^	BCMA	FHVH‐T, human	Auto	I	3–10 (6)	25	92	15	NR	24	8
NCT05066646 FUMANBA‐1^27^	BCMA	CT103A, human	Auto	Ib/II	3–23 (4)	103	96	NR (12 month PFS 78.8%)	NR	1	Not reported
NCT04309981 CARTBCMA‐HCB‐01 Interim analysis^28^	BCMA	ARI0002h, human	Auto	I/II	≥2	35	100	NR	NR	0	0
NCT03975907 LUMMICAR‐1^29^	BCMA	Zevor‐cel, human	Auto	II	3–15 (4)	102	92.8	NR	NR	6.9	0
NCT04155749 Frigault et al^30^	BCMA	CART‐ddBCMA, synthetic	Auto	I	3–16 (5)	37	100	NR after median 12.1 months	NR after median 12.1 months	3	6
NCT04093596 UNIVERSAL Part A^31^	BCMA	ALLO‐715, human	Allo	I	3–11 (5)	43	55.8	8 (mDOR)	NR	2	0
NCT04555551 Mailankody et al^32^	GPRC5D	MCARH109, human	Auto	I	4–14 (6)	17	71	N/a	N/a	6	12
NCT04674813 Part A Bal et al^33^	GPRC5D	CC‐95266	Auto	I	3–13 (4)	17	86	N/a	N/a	0	0
NCT05016778 POLARIS^34^	GPRC5D	OriCAR‐017	Auto	I	≥3	10	100	NR	NR	0	0
Xia et al^35^	GPRC5D	GPRC5D‐CAR, human	Auto	II	2–12 (4)	33	91	N/a	N/a	0	3
Zhou et al^36^	BCMA‐GPRC5D	Murine BCMA, human GPRC5D	Auto	I	3–5 (3)	21	86	NR	NR	0	0
Shi et al^37^	BCMA + CD19	Both murine	Auto	I	1	10	100	NR (70% at 2 years)	NR	0	0
Garfall et al^38^	BCMA + CD19	CART‐BCMA, huCART19 – both human	Auto	I	A: ≥3; B: 1	A: 10; B: 20	A: 100; B: 55	A: 5.2; B: 10.3	A: 15.9; B: NR	0	0
Du et al^39^	BCMA‐CD19	GC012F	Auto	I	2–9 (5)	29	93.1	38	NR	7	0
NCT04662099 Li et al^40^	BCMA‐CS1	Both murine	Auto	I/IIa	2–8 (4)	16	81	9	NR (1 year 73%)	6	0
NCT03287804 AUTO2 Lee et al^41^	BCMA‐TACI	APRIL	Auto	I	3–6 (5)	11	45.5	5	12.3	0	0
Mei et al^42^	BCMA‐CD38	BM38, human	Auto	I	2–9 (4)	23	87	17.2	NR (1 year 93%)	22	0
Tang et al^43^	BCMA‐CD38	Not specified	Auto	I	2–3 (3)	16	87.5	NR (1 year 69%)	NR (1 year 75%)	31	0
Zhang et al^44^	BCMA + CD38	Human BCMA, murine CD38	Auto	II	4–12 (8)	22	90.9	48.7% at 24 months	56.6% at 24 months	27.3	0

Abbreviations: LOT, lines of treatment; mDOR, medium duration of response; NR, not reached.

**TABLE 2:** Grading of common toxicities of cellular immunotherapy.

Toxicity		Grade 1: Mild	Grade 2: Moderate	Grade 3: Severe	Grade 4: Life‐threatening
CRS	Fever	Temp ≥38°C	Temp ≥38°C	Temp ≥38°C	Temp ≥38°C
			With		
	Hypotension	None	Not requiring vasopressors	Requiring a vasopressor with or without vasopressin	Requiring multiple vasopressors (excluding vasopressin)
			And/or		
	Hypoxia	None	Requiring low‐flow nasal cannula or blow‐by	Requiring high‐flow nasal cannula, facemask, non‐rebreather mask or Venturi mask	Requiring positive pressure (e.g., CPAP, BiPAP, intubation and mechanical ventilation)
ICANS	ICE score^a^	7–9	3–6	0–2	0 (unrousable)
	Depressed consciousness	Awakens spontaneously	Awakens to voice	Awakens only to tactile stimulus	Unrousable or requires vigorous/repetitive tactile stimuli to arouse
	Seizure	N/a	N/a	Any clinical seizure focal or generalised that resolves rapidly or non‐convulsive seizures on EEG that resolve with intervention	Life‐threatening prolonged seizure (>5 min); or repetitive clinical or electrical seizures without return to baseline in between
	Motor findings	N/a	N/a	N/a	Deep focal motor weakness such as hemiparesis or paraparesis
	Elevated ICP/cerebral oedema	N/a	N/a	Focal/local oedema on neuroimaging	Diffuse cerebral oedema on neuroimaging; decerebrate or decorticate posturing; cranial nerve VI palsy; papilloedema; or Cushing triad
MNT		Asymptomatic (clinical observation only)/ mild symptoms	Moderate symptoms	Severe symptoms	Life‐threatening symptoms
Anaemia		Hb < LLN (100 g/L)	Hb <100–80 g/L	Hb <80 g/L; transfusion indicated	Life‐threatening anaemia
Thrombocytopaenia		<LLN—75 × 10^9^/L	<75–50 × 10^9^/L	<50–25 × 10^9^/L	<25 × 10^9^/L
Neutropaenia		<LLN—1.5 × 10^9^/L	<1.5–1.0 × 10^9^/L	<1.0–0.5 × 10^9^/L	<0.5 × 10^9^/L
Lymphopaenia		<LLN—0.8 × 10^9^/L	<0.8–0.5 × 10^9^/L	<0.5–0.2 × 10^9^/L	<0.2 × 10^9^/L
Infection		Asymptomatic or mild symptoms	Moderate symptoms	Severe but not immediately life‐threatening	Life‐threatening consequences, urgent intervention needed

*Note*: CRS and ICANS as per the American Society for Transplantation and Cellular Therapy, others derived from National Cancer Institute Common Terminology Criteria for Adverse Events (NCI‐CTCAE) version 5.0.^45^, ^46^ Grade 5 = death related to adverse event.

^a^ ICE score = Immune effector cell‐associated encephalopathy score, based on 10‐point ICE screening tool.

Ciltacabtagene autoleucel (Cilta‐cel) has an scFv consisting of two llama heavy chains which bind to two distinct BCMA epitopes, resulting in high avidity against human BCMA.^50^ In CARTITUDE‐1 (median of six prior LOT), ORR was 97%, with 67% achieving sCR.^22^ Responses were durable, with an impressive median PFS of 34.9 months and median OS not reached.^51^ While study populations differ in patient and disease characteristics, these responses are remarkably superior to results seen with Ide‐cel and other products. In the phase III CARTITUDE‐4, where patients with one to three prior lines were enrolled, Cilta‐cel achieved CR/sCR in 73.1% of lenalidomide‐refractory patients, compared with 21.8% in the standard care group receiving chemotherapy alone.^24^ In Cohort B of the ongoing CARTITUDE‐2 (NCT04133636), patients received Cilta‐cel second line following early clinical relapse. ORR was 88.9% and median time to first response was only 0.9 months.^23^ A recently published real‐world study of Cilta‐cel included 236 RRMM patients, with median of six prior LOT, and a higher proportion of extra‐medullary disease and high‐risk cytogenetics than in CARTITUDE‐1. Fifty‐six per cent would not have CARTITUDE‐1 eligibility criteria. ORR was 89% and ≥CRR was 70%. Median PFS was not reached after median follow‐up of 13 months.^52^

The FDA approved Ide‐cel in 2021 and Cilta‐cel in 2022, for RRMM following ≥4 prior LOT; the EMA authorised both CARs soon thereafter (after ≥3 prior LOT).^53^, ^54^, ^55^ In 2024, in light of CARTITUDE‐4, the FDA and EMA approved Cilta‐cel for lenalidomide‐refractory RRMM following ≥1 prior LOT including a PI and IMiD.^56^, ^57^ In 2024, the FDA also approved Ide‐cel for triple‐class exposed RRMM following ≥2 prior LOT.^58^

FHVH‐T is a CAR with a fully human anti‐BMCA heavy‐chain variable domain, designed to reduce immunogenicity. In 25 RRMM patients, ORR was 92% and median PFS was 65 weeks. Surprisingly, humoral anti‐CAR responses were detected in 4/12 evaluated patients.^26^ Equecabtagene autoleucel (CT103A), another fully human BCMA CAR, was granted conditional approval in China in 2023 as a fourth‐line treatment. In the phase Ib/II FUMANBA‐1 trial, 11.4% had received previous BCMA CAR‐T. Impressively, ORR was 96% with CR/sCR at 74.3%; 12‐month PFS was 78.8%.^27^ In the ongoing CARTBCMA‐HCB‐01 study in RRMM, patients received ARI0002h, a humanised BCMA CAR‐T, in three fractions, followed by a booster dose after ≥100 days. Interim analysis showed 100% ORR.^28^

Frigault et al engineered CART‐ddBCMA, which has a unique, synthetic small stable protein (D‐Domain) as its binding domain, to reduce immunogenicity and improve cell surface stability. In a phase I study, 33 RRMM patients were treated with CART‐ddBCMA and achieved an ORR of 100% with 71% CR/sCR, and at median follow‐up of 12.1 months, 86% of evaluable patients had minimal residual disease (MRD) negativity.^30^ The pivotal phase II iMMagine‐1 study (NCT05396885) with this CAR (anitocabtagene autoleucel) is underway.

### GPRC5D

GPRC5D is an orphan G protein‐coupled receptor which is highly expressed by MM cells. Its signalling mechanism and function have not yet been identified.^59^ Expression is restricted to PCs, skin (hair follicles and eccrine glands), filiform papillae of the tongue and seminiferous tubules of the testis.^60^ Expression was also found to be specifically enriched in the inferior olivary nucleus of the brainstem.^32^

MCARH109 is a humanised, lentivirally transduced GPRC5D CAR‐T employing 4‐1BB costimulation. In a first‐in‐human dose escalation study, ORR was 12/17 (71%), reduced to 58% in patients not exceeding the maximum tolerated dose of 150 × 10^6^ CAR‐T cells. Importantly, 7/10 patients who relapsed following prior BCMA therapy responded. Of the 12 responses, 6 relapsed after 3–9 months. Grade 4 toxicities included CRS (one patient), ICANS (one patient) and macrophage activation syndrome (one patient); two patients had a grade 3 cerebellar disorder of unclear aetiology.^32^ In a phase II trial of another humanised GPRC5D CAR‐T, ORR was 91% and response was achieved in 9/9 patients who previously received BCMA CAR‐T therapy.^35^

Zhou et al conducted a phase I trial of the first bispecific BCMA‐GPRC5D CAR‐T cells. ORR for 21 RRMM patients was 86%, ≥CRR 62% and 81% had MRD negativity. There was no grade ≥ 3 ICANS or CRS. Median PFS and OS were not reached after median follow‐up of 5.8 months.^36^

### FcRH5

FcRH5 (CD307) belongs to a family of six genes of the immunoglobulin superfamily. Function is unknown.^61^ FcRH5 is expressed in all B cells with peak expression in mature B cells. Expression in MM cells is more than threefold higher than in non‐malignant PCs.^62^

Jiang et al generated FcRH5‐BCMA bispecific CAR‐T cells which showed cytotoxicity against MM cells expressing FcRH5 and BCMA alone and in combination. Most significantly, FcRH5‐BCMA CAR‐T demonstrated improved tumour infiltration and longer survival than FcRH5 or BCMA monospecific CAR‐T cells in a subcutaneous NCI‐H929 xenograft model.^63^

### CD19

CD19, a co‐receptor of the B‐cell receptor, is typically absent in terminally differentiated PCs and MM cells. Nevertheless, low‐level expression is seen in a minor subset of less mature MM cells and is associated with drug resistance, relapse and poor survival.^64^ CD19 may therefore be a desirable target.

Safety and efficacy of co‐administered BCMA and CD19 CAR‐T have been demonstrated in late‐relapsed MM and in high‐risk newly diagnosed (NDMM) post‐ASCT.^37^, ^38^ GC012F is a BCMA‐CD19 bispecific CAR‐T developed with the FastCAR‐T platform. In a phase I study, response rate and durability were excellent: 29 patients (median of five prior LOT) had ORR 93.1% and sCRR 82.8%. Median PFS was 38 months.^39^ Lee et al are currently evaluating a dual BCMA/persistent CD19 CAR strategy.^65^

Roex et al developed bispecific BCMA‐CD19 allo‐CAR NK, by co‐electroporating cells from the NK‐92 cell line with BCMA and CD19 CAR mRNA. Dual‐ and single‐CAR NK‐92 cells showed equivalent cytotoxicity *in vitro*.^66^

### CS1/signalling lymphocyte activation molecule family member 7 (SLAMF7)

CS1 (also known as SLAMF7) is involved in immune regulation. It is expressed by dendritic cells, monocytes and lymphocytes, and upregulated in MM cells, in which it promotes cell proliferation and growth.^67^

Li et al constructed bispecific CS1‐BCMA CAR‐T cells, incorporating novel murine anti‐CS1 scFv and anti‐BCMA scFv in tandem, with 4‐1BB costimulation. In a phase I/IIa trial, ORR was 13/16 (81%).^40^

### CD229

CD229 is another member of the signalling lymphocyte activation molecule (SLAM) family. Expression is restricted to B and T cells and it is universally and strongly expressed by MM cells. Silencing of CD229 causes spontaneous MM cell apoptosis.^68^ Efficacy of CD229 CAR‐T has been demonstrated in preclinical models, both as a single‐targeted therapy and with a CD229‐BCMA bicistronic CAR‐T.^69^, ^70^

### Semaphorin‐4A (SEMA4A)

SEMA4A is a cell surface protein involved in embryonic and pathological angiogenesis, fine‐tuning of the immune response and retinal function.^71^ It is expressed by MM cells at a higher level than BCMA and SLAMF7. Loss of SEMA4A leads to MM cell apoptosis.^72^ A humanised SEMA4A CAR‐T demonstrated potent and specific anti‐MM activity in vitro.^73^

### Transmembrane activator, calcium modulator and cyclophilin ligand interactor (TACI)

TACI, like BCMA, is a member of the TNFR superfamily that is almost exclusively expressed by PCs, and upregulated by most MM cells.^74^ BCMA and TACI have two primary natural ligands: B‐cell activating factor (BAFF) and a proliferation‐inducing ligand (APRIL).^6^ APRIL ligand as a CAR was evaluated in the phase I AUTO2 trial but showed limited efficacy with ORR of 45.5%, median PFS of 5 months and median OS of 375 days. The APRIL CAR demonstrated in vitro functional deficiencies including reduced target binding, reduced IL‐2 secretion and impaired interferon signalling compared with other BCMA CARs.^75^ A trimeric APRIL‐based (TRIPRIL) CAR has been developed to enhance binding to both BCMA and TACI and is currently being evaluated in a clinical study (NCT05020444).^76^

### CD38

CD38 has high, uniform expression on MM cells. Three trials involving dual targeting of CD38 and BCMA have been conducted in China. BM38 CAR is a humanised bispecific BCMA‐CD38 CAR. In a phase I study, 23 RRMM patients had ORR of 87% including 52% sCR. Responses were durable with median PFS of 17.2 months.^42^ A second bispecific BCMA‐CD38 CAR‐T product showed ORR of 87.5% with 81% sCR.^43^ In both trials, grade ≥ 3 CRS was common (22% and 31% including one death respectively). In a third trial, 22 RRMM patients with median of eight prior LOTs received humanised BCMA CAR‐T cells in combination with murine anti‐CD38 CAR‐T cells, and achieved an ORR of 90.9%, 24‐month PFS rate of 48.7% and OS rate of 56.6%. Grade 3 CRS occurred in 27.3%.^44^

In dual‐split CD38‐CD138 CAR‐T cells, the stimulatory and costimulatory signals were split into two separate stimulatory and costimulatory CARs (sCAR and cCAR) to increase specificity for MM cells. The most optimal combination *in vitro* was a low‐affinity CD138sCAR combined with a high‐affinity CD38cCAR. This combination also demonstrated efficacy against MM cells with reduced CD38 expression following daratumumab pretreatment.^77^

## CAR‐T TOXICITIES

In addition to CRS and ICANS (discussed in the Introduction), other toxicities to CAR‐T therapy include prolonged cytopaenias, with median time to recovery of grade 3–4 cytopaenias of 2 months following BCMA CAR‐T therapy.^22^ Age and cumulative treatment‐related toxicity appear to be significant contributing factors.^78^ Infection is also very common. In 55 patients treated with BCMA CAR‐T, 47 infections were diagnosed in 29 patients (53%); 6% were severe and 2% fatal.^79^ Infection risk is compounded by treatment‐associated lymphopaenia and hypogammaglobulinaemia. Lancman et al observed 90% reduction in grade 3–5 infections during periods of treatment with IV immunoglobulin (IVIG) following BCMA‐targeted BiTE therapy; IVIG is likely to provide valuable prophylaxis following BCMA CAR‐T as well.^80^ IVIG is therefore administered monthly until resolution of hypogammaglobulinaemia.

Movement and neurocognitive toxicities (MNTs) occurred in 5% of patients in CARTITUDE‐1.^53^ MNTs manifest after a period of recovery from CRS and/or ICANS. MNTs are associated with high tumour burden, grade ≥ 2 CRS, any ICANS and high CAR‐T cell expansion/persistence.^81^ In CARTITUDE‐4, following strategies to reduce baseline tumour burden with bridging therapy, together with more aggressive, early management of CRS and ICANS, incidence of MNTs decreased to one grade 1 case (0.2%).^24^ Management approaches include corticosteroids, plasmapheresis and IVIG; Graham et al also reported a case of rapid improvement following cyclophosphamide.^81^, ^82^

In view of GPRC5D expression in the skin and tongue, GPRC5D CAR‐T therapy can cause time‐limited on‐target, off‐tumour toxicities which include nail changes (12%–65%), skin changes/rash (3%–18%) and dysgeusia/dry mouth (12%). These were all grade 1 apart from one patient who developed grade 2 desquamation of palms and soles.^32^, ^33^, ^35^ The exact cause of the grade 3 persistent cerebellar disorders seen in two recipients of MCARH109 is unclear but could relate to expression of GPRC5D in the inferior olivary nucleus.^32^

It is important to consider whether dual‐antigen targeting confers additional toxicity. With the dual BCMA‐CD19 targeting approaches discussed above, grade 3–4 CRS was identified in total of 2/69 patients (3%) and there were no grade 3–4 ICANS.^37^, ^38^, ^39^ No grade 3–4 CRS or ICANS were observed in 21 patients who received bispecific BCMA‐GPRC5D CAR‐T cells.^36^ In contrast, grade ≥ 3 CRS was more prevalent with dual BCMA‐CD38 targeting at 22%–27.3% in the three trials discussed, including one death.^42^, ^43^, ^44^

## OVERCOMING CAR‐T RESISTANCE

Despite impressive response rates seen with CAR‐T in MM, including high rates of CR/sCR, the majority still relapse. The disease kinetics that mediate resistance to CAR‐T are poorly understood. Work to identify and circumvent mechanisms of resistance has predominantly focused on BCMA. Mechanisms can be categorised into tumour‐intrinsic factors, host factors and CAR‐T factors.

- *Tumour‐intrinsic factors*: These include higher disease burden, high‐risk cytogenetics and extra‐medullary disease.^83^ BCMA is important for PC function and unlike CD19, where loss or downregulation is seen in up to one‐third of patients who relapse following CD19 CAR‐T, BCMA loss is rare.^84^ In KarMMa, BCMA expression was lost in 3/71 (4%) of patients who progressed following Ide‐cel.^19^ In one of the cases, homozygous deletion of *TNFRSF17* (BCMA) on chromosome 16 was subsequently confirmed.^85^ In another case report, Samur et al describe biallelic BCMA loss involving deletion of one allele and a mutation that engendered an early stop codon on the other allele.^86^ Mutational events appear a more common mechanism of resistance following BCMA‐directed BiTE therapy. Lee et al analysed 14 cases of relapse following teclistamab or elranatamab, and identified biallelic *TNFRSF17* deletion in one case, as well as functional epitope loss secondary to non‐truncating, missense mutations or in‐frame deletions in five cases, despite evidence of surface BCMA expression. They postulated that the repeated administration of BiTEs exerts a longitudinal selective pressure that is avoided by the single administration of BCMA CAR‐T.^87^ Perica et al observed that while BCMA loss is uncommon following BCMA CAR‐T, resistance/relapse is associated with reduction in BCMA expression below the threshold necessary for optimal CAR‐T cell functioning.^88^ Dual‐antigen targeting approaches, discussed above, are intended to thwart the risk posed by BCMA loss in order to improve depth and duration of response.
  BCMA is cleaved from the MM cell surface by the γ‐secretase complex, releasing soluble BCMA and reducing target density (Figure 1). Concurrent use of a γ‐secretase inhibitor (GSI) enhanced anti‐tumour efficacy of BCMA CAR‐T in an MM murine model.^89^ In a first‐in‐human trial, an oral GSI (JSMD194) was co‐administered with BCMA CAR‐T. Following JSMD194, BCMA antigen binding capacity increased by 20‐fold in seven heavily pretreated patients. ORR was 100% among the six assessable patients (one patient died on day +33 in context of CRS and fungal infection).^90^
  In contrast to BCMA CAR‐T, antigenic escape appears to be a key mechanism of resistance to GPRC5D CAR‐T, with loss/decrease of GPRC5D observed in all six patients who relapsed following MCARH109.^35^ Derrien et al used a single‐nucleus multiomic strategy to analyse three cases of relapse following GPRC5D‐targeted BiTE therapy and identified two resistance mechanisms: biallelic *GPRC5D* inactivation and long‐range epigenetic silencing of its promoter and enhancer regions.^91^
  MM cells express the inhibitory ligands PD‐L1 and PD‐L2 (ligands for PD‐1), MHC class II (ligand for LAG‐3) and galectin‐9 (ligand for TIM‐3), which enable immune escape.^83^ PD‐1 knock‐out significantly enhanced anti‐MM activity of cytotoxic T cells in vitro and in a murine xenograft model.^92^ Trials of PD‐1 blockade with nivolumab or pembrolizumab following BCMA CAR‐T are ongoing (NCT04205409 and NCT05191472). Yuti et al generated anti‐BCMA‐CAR5‐T cells, which secrete anti‐PD‐L1 scFv blockade molecules. They demonstrated superior anti‐tumour efficacy in vitro, compared with non‐PD‐L1‐blocking CAR‐T cells.^93^ Wang et al registered a phase II trial investigating BCMA‐PD1‐CART, a TRUCK which secretes a PD‐1 Fc fusion protein (NCT04162119).
  Melnekoff et al observed significantly upregulated expression of the anti‐apoptotic genes MCL‐1, FOSB and JUND following relapse after Ide‐cel.^94^ Targeting of the MCL‐1/BCL‐2 axis may therefore be appropriate to treat selected relapsed patients. ORR for patients who relapsed following BCMA CAR‐T and received first‐line salvage with venetoclax was 66.7%.^95^
  Interferon‐γ (IFN‐γ) plays a vital role in orchestrating anti‐tumour immune responses. Larson et al generated IFN‐γ receptor 1 knock‐out (IFNγR1‐KO) tumours in a variety of cancer cell lines. They found that disrupted IFN‐γ receptor signalling promoted resistance to CAR‐T cell cytotoxicity in vitro and in vivo in glioblastoma and other solid tumours, but not in MM or lymphoma.^96^
- *Host factors*: These include the TME and T‐cell fitness. Stromal cells in the TME produce extra‐cellular matrix proteins that limit trafficking of tumour‐infiltrating T cells, via activation of TGF‐β.^97^ The TME also recruits Treg cells, myeloid‐derived suppressor cells and tumour‐associated macrophages, which express immunosuppressive cytokines including TGF‐β and IL‐4.^98^ Armoured B2ARM CAR‐T cells co‐express a BCMA CAR and TGF‐β dominant‐negative receptor II, endowing resistance to TGF‐β. In preclinical models, B2ARM CAR‐T cells demonstrated superior persistence and anti‐tumour function compared with non‐armoured CAR‐T cells.^99^
  Anti‐MM treatment impairs baseline T‐cell fitness. Heavy pretreatment decreases the CD4/CD8 T cell ratio and the frequency of early memory T cells in the leukapheresis product, hindering CAR‐T expansion and persistence.^100^ T cells from resistant patients expressed higher levels of the inhibitory receptors PD‐1, LAG‐3 and TIGIT; blockade of TIGIT enhanced MM cell death in vitro.^101^ It is therefore possible that earlier leukapheresis will improve CAR‐T efficacy. Dubnikov Sharon et al evaluated clinical outcome of CD19 CAR‐T cells manufactured from earlier leukapheresis (at first relapse) compared with standard leukapheresis (at second relapse or beyond) in diffuse large B‐cell lymphoma (DLBCL). The early group demonstrated increased percentage of naive T cells, increased T‐cell functionality in vitro and lower exhaustion profile. This did not translate into significantly improved clinical outcome, although a trend towards better PFS and OS was seen.^102^ CAR‐T cell fitness may also be improved by use of healthy donor (HD)‐derived allogeneic CAR‐T cells (see ‘Allogeneic CAR‐T’ below).
- *CAR‐T factors*: Efficacy of CAR‐T therapy in MM is influenced by several aspects of CAR‐T manufacture. CARs with fully humanised or synthetic binding domains are engineered to reduce immunogenicity. Compared with CD28 costimulation, CD19 CARs employing 4‐1BB had slower activation but superior persistence with better maintenance of memory phenotype.^103^ The BCMA CAR‐T product bb21217 incorporates the same CAR construct as Ide‐cel, with the addition of a PI3K inhibitor (bb007) during ex vivo culture, to enrich memory‐like T cells.^104^
  The bespoke manufacture of autologous CAR‐T cells is costly and takes up to 6 weeks, during which time there is risk of disease progression. Manufacturing time can be reduced to 22–36 h with the use of FastCAR‐T platforms such as the CliniMACS Prodigy® (Miltenyi Biotec) and the Cocoon® (Lonza).^25^, ^39^
  Melnekoff et al observed significant expansion of non‐CAR CD8^+^ T cells with effector memory immunophenotype 4 weeks after Ide‐cel, suggesting that IMiDs, which provide a costimulatory signal to T cells, may augment CAR‐T efficacy.^94^ Indeed, lenalidomide potentiated BCMA CAR‐T activity in vitro and significantly prolonged survival in a murine MM model.^105^ In a case report, a patient with IgD‐λ MM who progressed after multiple LOT including ASCT and two prior BCMA CAR‐T products was treated with humanised BCMA CAR‐T combined with 25 mg lenalidomide once daily. He achieved VGPR which was maintained for more than 8 months.^106^ Prospective studies are required to evaluate safety and efficacy of co‐administration of IMiDs.
  Wachsmann et al recently proposed combining BCMA CAR‐T with T‐cell receptor (TCR)‐engineered T cells to prevent immune escape. Unlike CARs, TCRs recognise peptides presented in the context of human leukocyte antigens (HLA). They transduced T cells to express a TCR targeting a peptide derived from BOB1, a transcription factor (TF) cofactor essential for MM cell survival. Single‐antigen targeting resulted in immune escape and proliferation of tumour cells in vitro and in vivo, whereas dual‐antigen targeting completely cleared MM cell populations.^107^ Simultaneous targeting using HLA‐dependent and HLA‐independent recognition modes may therefore be complementary.
  CD38 is weakly expressed by T cells and therefore CD38 CAR‐T cells are fraught with the potential for fratricide. To overcome this, BM38 CAR‐T cells were manufactured with a high affinity of anti‐CD38 scFv and reduced affinity of anti‐CD38 scFv, to selectively eliminate CD38^++^ MM cells but spare CD38^+^ T cells.^42^

## ALLOGENEIC CAR‐T

In addition to the risk of disease progression during the 6‐week manufacture of auto‐CAR‐T, manufacturing failure occurred in 18% of patients in CARTITUDE‐1.^22^ Allo‐CAR‐T can be manufactured from young HDs, produced in large batches and cryopreserved, allowing immediate ‘off‐the‐shelf’ availability with decreased cost.^108^ Metelo et al compared efficacy of HD‐derived and MM‐derived BCMA CAR‐T cells in vitro. The HDs had higher T‐cell counts, CD4/CD8 ratio, naive T‐cell phenotype and increased proportion of central memory cells. HD‐derived T cells had superior transduction rate, superior cytotoxicity and greater expansion compared with late‐relapsed MM‐derived CAR‐T cells.^109^

αβ T cells constitute 95% of peripheral blood T cells.^110^ Allo‐CAR‐αβ T cells require gene editing to inactivate the endogenous TCRα/β, to prevent alloreactivity in the form of graft‐versus‐host disease (GvHD). To date, allo‐CAR‐αβ T‐cell therapies targeting BCMA and CS1 have been trialled in humans and are reviewed here.

Sommer et al manufactured an allo‐BCMA CAR which reduced tumour burden in a murine model.^111^ In the first‐in‐human phase I UNIVERSAL trial, RRMM patients received the allo‐BCMA CAR‐T ALLO‐715 +/− the GSI nirogacestat (NCT04093596). ALLO‐715 contains an integrated, recombinant lentiviral vector encoding a humanised BCMA CAR with a CD20 off‐switch and 4‐1BB costimulation. *TRAC* (TCRα constant) and *CD52* were knocked out by transcription activator‐like effector nucleases (TALENs) to obviate GvHD and confer resistance to alemtuzumab respectively. Interim results demonstrated 55.8% ORR and median duration of response (mDOR) of only 8.3 months. Toxicities were significant: 23.3% had grade ≥ 3 infection including 7% grade 5. Twenty‐eight per cent had grade 1–2 CMV reactivation and 5% had grade ≥ 3. No GvHD was observed.^31^ Other allo‐BCMA CAR‐T trials include ALLO‐605 (IGNITE study, NCT05000450—terminated prematurely, data not yet published) and the ongoing P‐BCMA‐ALLO1 (NCT04960579).

UCARTCS1 is a CS1 allo‐CAR‐T, in which TALENS were used to disrupt the genes encoding TCRα and CS1, to obviate GvHD and CS1‐mediated fratricide respectively. UCARTCS1 showed specific and dose‐dependent anti‐MM activity in preclinical models.^112^ In preliminary results for MELANI‐01 (NCT04142619), a first‐in‐human phase I dose escalation trial, 2/5 patients responded; however, both subsequently died (one from organising pneumonia and the second from haemorrhagic pancreatitis in the context of grade 4 CRS, HLH, disseminated mucormycosis and pseudomonal pneumonia). After a clinical hold by the FDA, the study resumed with protocol updates to mitigate the risks from prolonged lymphopaenia and CRS.^113^

Kinder et al validated an allogeneic hypoimmune (HIP) GPRC5D CAR‐T. Manufacture involved disruption of the *TCR*, *B2M* and *CIITA* genes to prevent GvHD, and lentiviral transduction to express a humanised GPRC5D CAR and CD47, which promotes CAR‐T persistence by protecting from innate immune system killing. The HIP GPRC5D CAR‐T cells showed in vitro and in vivo efficacy in line with a clinically validated GPRC5D CAR‐T.^114^

γδ T cells, natural killer (NK) cells and invariant natural killer T cells (iNKT) cells recognise antigens in an HLA‐independent fashion and do not require TCR gene editing.^115^ γδ T cells are a small group of effector T cells, comprising 1%–5% of circulating CD3+ T cells. Zhang et al electroporated Vγ9Vδ2 T cells (a subset of γδ T cells) with BCMA CAR‐encoding mRNA. The resultant CAR‐Vγ9Vδ2 T cells reduced tumour burden and significantly prolonged survival in murine MM models.^116^

NK cells play an important role in immune surveillance of cancer and viral infections.^117^ CARs arm NK cells with cancer‐specific cytotoxicity; however, NK cell manipulation can downregulate homing receptors required for trafficking, and CAR‐NK cells need modification to overcome this.^115^ Ng et al electroporated NK cells with mRNA encoding a BCMA CAR and CXCR4. The CAR‐NK cells significantly reduced tumour burden and extended survival in xenograft murine MM models. Furthermore, transgenic CXCR4 expression promoted CAR‐NK cell migration to the bone marrow, compared with non‐CXCR4‐transfected CAR‐NK cells.^118^ FT576 is an iPSC‐derived BCMA CAR‐NK product which demonstrated durable antitumour activity in an MM murine model.^119^ A phase I trial of FT576 +/− daratumumab is in progress (NCT05182073).

CAR NK cells have also been developed to target CS1, GPRC5D and CD38. CS1 CAR NK was generated from NK‐92 cells. In a murine model, the product suppressed growth of human IM9 MM cells and significantly prolonged survival.^120^ Bicistronic BCMA‐GPRC5D allo‐CAR NK cells reduced relapse and improved survival compared with single‐targeting CAR NK cells in a xenograft murine model.^121^ Stikvoort et al manufactured CD38 CAR NK from KHYG‐1 cells, an immortal NK cell line. It showed excellent expansion and effective CD38‐dependent cytotoxicity in vitro and in a xenograft murine model. Importantly, it was also effective against primary MM cells from daratumumab‐refractory patients.^122^

iNKT cells have an invariant TCR that interacts with altered glycolipids presented by CD1d, a non‐polymorphic MHC‐I‐like molecule. Poels et al demonstrated CAR‐dependent cytotoxicity of both BCMA and CD38 CAR iNKT cells against the UM9 MM cell line, without compromising their inherent cytotoxic functioning.^123^ O'Neal et al recently demonstrated significant anti‐MM activity of human BCMA CAR iNKT cells in a xenograft murine model. CAR iNKT cells induced less IL‐6 than CAR‐T cells, suggesting reduced potential for CRS.^124^

## CONCLUSION AND FUTURE DIRECTIONS

BCMA CAR‐T has achieved hitherto unseen outcomes in heavily pretreated RRMM patients and in early relapse. Nevertheless, the prospect of long‐term cure remains elusive. Strategies to overcome resistance include γ‐secretase inhibition and arming the CAR‐T to inhibit PD‐L1 or TGF‐β. Antigen escape can be mitigated by multiantigen targeting; the most impressive results to date are for bispecific BCMA‐CD19 CAR‐T; however, many other potential target combinations require evaluation.^39^

BCMA CAR‐T is the most expensive MM treatment to date. Ide‐cel and Cilta‐cel currently cost ~0.5 million USD per infusion.^125^ Cost‐effectiveness will improve with prolonged PFS. As discussed, manufacturing time can be expedited to 22–36 h with FastCAR‐T platforms.

CAR‐T cell fitness, cost and availability can be improved with the use of allogeneic products. The early trial results for ALLO‐715 and UCARTCS1 reveal no GvHD but limited duration of response and significant risk of infection.^31^, ^113^ Further research is needed to optimise the safety and efficacy of allo‐CAR treatments.

The role of BCMA CAR‐T as a front‐line treatment in NDMM is yet to be elucidated. Ongoing trials include phase I KarMMa‐4, a single‐arm study of Ide‐cel following four cycles of induction therapy in patients with high‐risk NDMM (NCT04196491). *EM*agine/CARTITUDE‐6 is a phase III randomised study comparing Cilta‐cel with ASCT in transplant‐eligible patients with NDMM, following induction with daratumumab, velcade, lenalidomide and dexamethasone (DVRd) (NCT05257083). Meanwhile, CARTITUDE‐5 is a phase III randomised study comparing Cilta‐cel with Rd in patients with NDMM not intended for ASCT, following induction with VRd (NCT04923893). CAR‐PRISM is a phase II trial investigating Cilta‐cel at an even earlier stage, in the setting of high‐risk smouldering MM (NCT05767359).

Teclistamab is a BiTE targeting BCMA and CD3 and was NICE approved as fourth‐line treatment for RRMM in October 2024.^126^ Talquetamab, a GPRC5DxCD3 BiTE, is FDA approved as fifth‐line treatment and has received a conditional marketing authorisation from the EMA.^127^, ^128^ Other BiTEs include elranatamab and linvoseltamab (targeting BCMAxCD3) and cevostamab (FcRH5xCD3).^7^ With the simultaneous emergence of CAR‐T therapy and BiTEs, it is important to consider the ideal sequencing of these immunotherapies. BiTEs are cheaper, readily available ‘off‐the‐shelf’ and require less resources to manufacture and deliver than CAR‐T. Lymphodepletion is not required and toxicity is typically less severe, with lower frequency and grade of CRS and ICANS. These factors make BiTEs a promising option for frailer patients. However, clinical outcomes of BiTEs are inferior to CAR‐T. In the phase I–II MAJESTEC‐1 study of 165 RRMM patients, teclistamab achieved ORR of 63% and median PFS of 11.3 months after median of five previous LOT, comparing unfavourably with the majority of CAR‐T outcomes reviewed here.^129^ While CAR‐T cells are transduced and expanded in vitro, BiTE efficacy is more reliant on underlying T‐cell fitness. BiTEs have a shorter half‐life and require ongoing treatment, prolonging the risks of cytopaenias and hypogammaglobulinaemia and necessitating long‐term monthly IVIG. For these reasons, it appears that CAR‐T should preferentially be delivered earlier than BiTEs in the treatment journey of fit patients. In the MagnestisMM studies, patients previously exposed to BCMA CAR‐T (28% of whom were refractory) who were treated with elranatamab had ORR of 52.8%, compared with 61% in CAR‐T naive patients.^130^, ^131^ There is, therefore, a role for BCMA x CD3 BiTEs in the setting of BCMA CAR‐T relapse; however, it is not clear how this approach would compare with targeting a different MM antigen with CAR‐T, BiTE or other therapy. Further studies are required to establish the optimal sequence of ASCT, CAR‐T, BiTEs and other treatments in MM. Notwithstanding, treatment decisions will ultimately be based on accessibility, with NICE approval awaited for BCMA CAR‐T.

Following approval of novel cellular immunotherapies, patient access presents significant new challenges. More Good Manufacturing Practice (GMP)‐approved facilities are required, together with increased resources to manage patients throughout the treatment journey. Training is required in supportive care and prompt diagnosis and management of CRS, ICANS and other toxicities. CAR‐T therapy is a rapidly developing field with potential to transform outcomes in RRMM, and healthcare centres need to rise to these challenges.

## AUTHOR CONTRIBUTIONS

EM undertook literature review and wrote the manuscript; RB reviewed and edited the manuscript.

## CONFLICT OF INTEREST STATEMENT

EM: None declared; RB: Received research funding from Servier and Allogene.
